# Bypass of Activation Loop Phosphorylation by Aspartate 836 in Activation of the Endoribonuclease Activity of Ire1

**DOI:** 10.1128/MCB.00655-16

**Published:** 2017-07-28

**Authors:** Michael C. Armstrong, Sergej Šestak, Ahmed A. Ali, Hanan A. M. Sagini, Max Brown, Karen Baty, Achim Treumann, Martin Schröder

**Affiliations:** a Durham University, Department of Biosciences, Durham, United Kingdom; b Biophysical Sciences Institute, Durham University, Durham, United Kingdom; c North East England Stem Cell Institute (NESCI), Life Bioscience Centre, International Centre for Life, Newcastle upon Tyne, United Kingdom; d Slovak Academy of Sciences, Institute of Chemistry, Department of Glycobiology, Bratislava, Slovakia; e Molecular Biology Department, National Research Center, Cairo, Egypt; f NEPAF, Newcastle upon Tyne, United Kingdom

**Keywords:** endoplasmic reticulum, Ire1, unfolded protein response, phosphorylation, protein kinase, phosphorylation-independent RD kinase, stress response

## Abstract

The bifunctional protein kinase-endoribonuclease Ire1 initiates splicing of the mRNA for the transcription factor Hac1 when unfolded proteins accumulate in the endoplasmic reticulum. Activation of Saccharomyces cerevisiae Ire1 coincides with autophosphorylation of its activation loop at S840, S841, T844, and S850. Mass spectrometric analysis of Ire1 expressed in Escherichia coli identified S837 as another potential phosphorylation site *in vivo*. Mutation of all five potential phosphorylation sites in the activation loop decreased, but did not completely abolish, splicing of *HAC1* mRNA, induction of *KAR2* and *PDI1* mRNAs, and expression of a β-galactosidase reporter activated by Hac1^i^. Phosphorylation site mutants survive low levels of endoplasmic reticulum stress better than *IRE1* deletions strains. *In vivo* clustering and inactivation of Ire1 are not affected by phosphorylation site mutants. Mutation of D836 to alanine in the activation loop of phosphorylation site mutants nearly completely abolished *HAC1* splicing, induction of *KAR2*, *PDI1*, and β-galactosidase reporters, and survival of ER stress, but it had no effect on clustering of Ire1. By itself, the D836A mutation does not confer a phenotype. These data argue that D836 can partially substitute for activation loop phosphorylation in activation of the endoribonuclease domain of Ire1.

## INTRODUCTION

Accumulation of unfolded proteins in the endoplasmic reticulum (ER) activates the bifunctional transmembrane protein kinase-endoribonuclease Ire1 (1, 2). In ER-stressed cells, Ire1 oligomerizes (3, 4) and concentrates in clusters at the ER membrane (5, 6) independent of its protein kinase activity (6, 7). In the yeast Saccharomyces cerevisiae, activated Ire1 cleaves the mRNA for the transcription factor *HAC1* to initiate removal of a 252-nucleotide intron from *HAC1* mRNA (8–11) that inhibits translation of unspliced *HAC1* (*HAC1*^u^) mRNA (12). Ligation of the 5′ and 3′ exons of *HAC1* mRNA by tRNA ligase produces spliced *HAC1* mRNA (*HAC1*^i^) (13), which is readily translated. Hac1^i^ induces transcription of genes encoding ER-resident molecular chaperones and protein foldases, such as *BiP*/*KAR2* and *PDI1*, to alleviate the unfolded protein burden in the ER (14, 15). Many Hac1^i^ target genes contain a short promoter element, termed the unfolded protein response element (UPRE) (16), to which Hac1^i^ binds as a heterodimer with the transcription factor Gcn4 (17).

**FIG 1:**
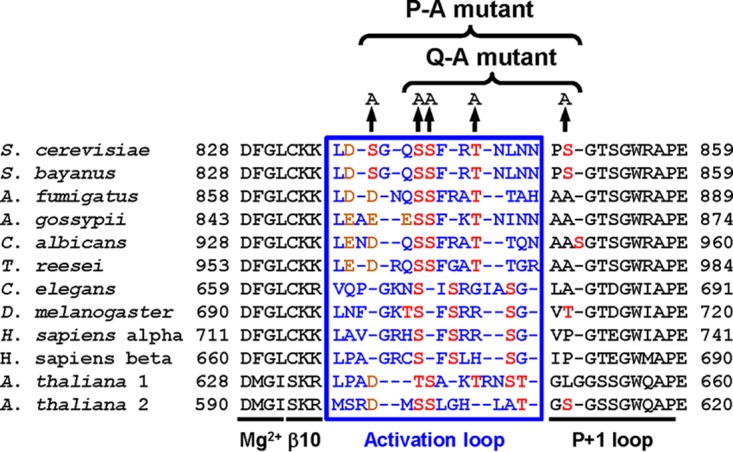
Sequence alignment of the activation segment of Ire1. The species used and their GenBank accession numbers are the following: Arabidopsis thaliana 1, NP_568444; A. thaliana 2, NP_565419; Ashbya gossypii, NP_984389; Aspergillus fumigatus, XP_749922; Caenorhabditis elegans, AAL30828; Candida albicans, XP_717606; Drosophila melanogaster, NP_001097839; Homo sapiens α, NP_001424; H. sapiens β, NP_150296; Saccharomyces cerevisiae, NP_011946; and Trichoderma reesei, AAP92915. The sequence for Saccharomyces bayanus Ire1 was obtained from the Saccharomyces Genome Database. Amino acids in the activation loop are shown in blue, potential phosphoacceptor sites are in red, and aspartic and glutamic acid are in orange.

The N-terminal lobe of protein kinases harbors an activation segment whose start and endpoints are defined by the conserved amino acid sequence motifs DFG and APE in S. cerevisiae Ire1. The activation segment encompasses the Mg^2+^-binding loop, a short β strand, β9 (β10 in Ire1 [18]), the activation loop, and the *P* + 1 loop (Fig. 1) (19). Phosphorylation in the activation loop activates many protein kinases (20). Phosphorylation-dependent protein kinases often display an invariant arginine (R796 in Ire1) that precedes the catalytic aspartate (D797 in Ire1) (20). Crystallographic examination of active conformations of these RD kinases has revealed that phosphoamino acids in the activation loop are in contact with a basic pocket formed by the invariant arginine, a basic amino acid located in β9 (K833 on β10 in Ire1), and often a third basic amino acid located in helix αC in the N-terminal lobe (19, 20). The invariant arginine R796, preceding the catalytic aspartate D797 and a lysine in β10, K833, have been conserved throughout evolution in Ire1 (Fig. 1). Mass spectrometric mapping of tryptic peptides revealed four potential phosphorylation sites in Ire1, S840, S841, T844, and S850 (18). Mutational analyses illustrate the importance of phosphorylation of Ire1 *in vivo*. An S840A S841A double mutant displayed a large defect in induction of both *KAR2* and *PDI1* mRNAs (3), while *HAC1* splicing by an S840A S841A T844A triple mutant and survival of ER stress by an S840A S841A T844A S850A quadruple mutant were severely decreased (18, 21). In addition, phosphorylation of human IRE1α increased its RNase activity toward a short, fluorescently labeled stem-loop substrate ∼100-fold (22).

However, *HAC1* splicing in cells expressing S840A-S841A-T844A-Ire1 nevertheless reached ∼20% of the level of *HAC1* splicing in cells expressing WT Ire1 (21), which suggests that the S840A S841A T844A mutant transduces a weak ER stress signal. In addition, mutation of catalytic amino acids in the protein kinase active site, for example, in D828A and D797A K799A mutants, decreases, but does not abolish, processing of *HAC1* mRNA by Ire1 (7, 21). Finally, addition of the nonphosphorylatable ATP analogue 1-*tert*-butyl-3-naphthalen-1-ylmethyl-1*H*-pyrazolo[3,4-*d*]pyrimidin-4-ylemine to cells expressing protein kinase-deficient mutants restored the ability to process *HAC1* mRNA (23). These observations suggest that activation loop phosphorylation is dispensable for at least partial activation of Ire1. The purpose of this study was to elucidate mechanisms that allow Ire1 to transduce an ER stress signal in the absence of phosphorylation in its activation segment. Our work identifies an additional potential phosphorylation site in the activation segment, S837. A mutant lacking all phosphorylation sites in the activation segment, including S837, still transduces a weak ER stress signal. The ability to transduce this weak ER stress signal requires the presence of an aspartate, D836, in the activation loop.

## RESULTS

### S837 is a potential autophosphorylation site.

**FIG 2:**
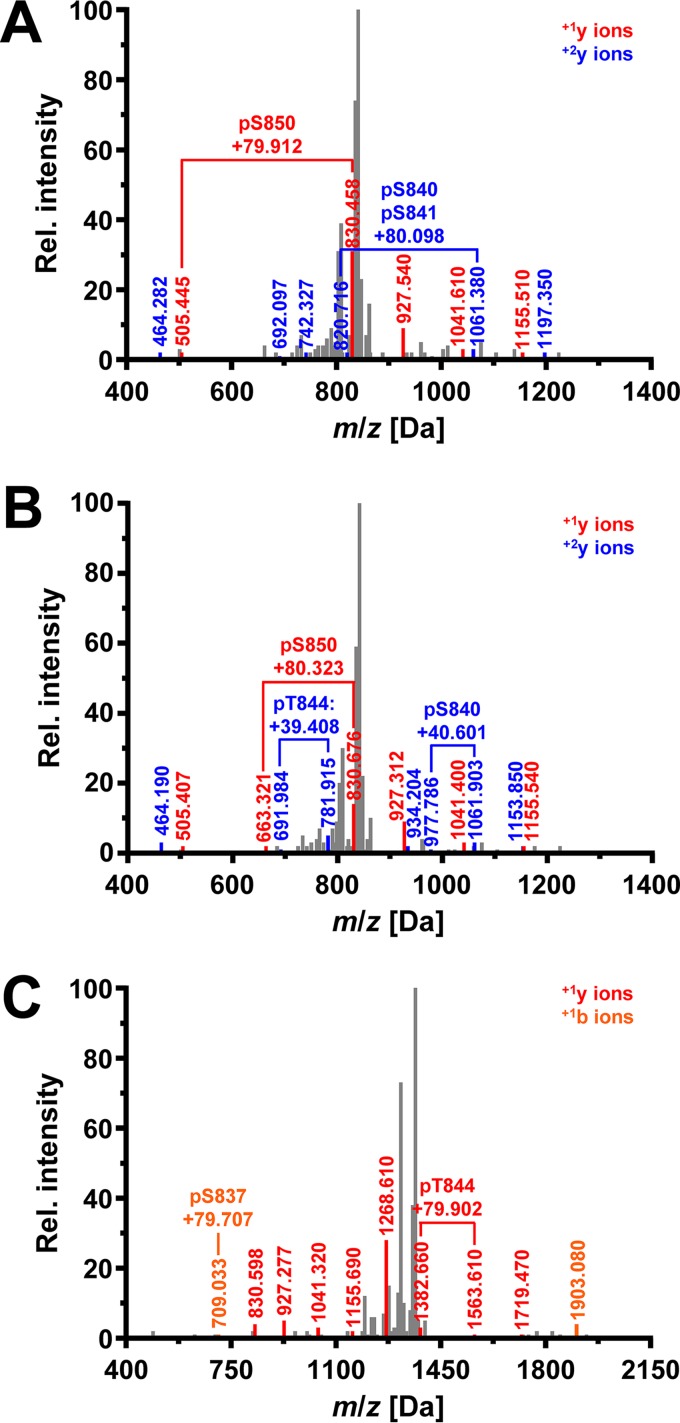
Ire1 autophosphorylates at S837. (A) Fragmentation spectrum 1976 for the peptide ^835^LDSGQpSpSFRTNLNNPpSGTSGWR^856^ derived from WT Ire1. Detected mass-to-charge ratios for the ^+1^y (red) and ^+2^y (blue) ion series are shown. Brackets highlight fragmentations that are explained by the presence of phosphoryl groups. The difference between the observed mass-to-charge ratio and the monoisotopic mass-to-charge ratio for the unphosphorylated ions are indicated. (B) Fragmentation spectrum 2031 for the peptide ^835^LDSGQpSSFRpTNLNNPpSGTSGWR^856^ derived from WT Ire1. (C) Fragmentation spectrum 1462 for the peptide ^834^KLDpSGQp(SS)FRpTNLNNPp(SGTS)GWR^856^ derived from L745A-Ire1. Detected mass-to-charge ratios for the ^+1^y (red) and ^+1^b (orange) ion series are shown. In addition, one phosphoryl group is bound to S840 or S841 and another phosphoryl group to S850, T852, or S853.

To identify autophosphorylation sites in the activation segment of Ire1, we expressed the cytosolic portion of Ire1 starting at Q556 as an N-terminal glutathione *S*-transferase fusion protein in Escherichia coli, purified it by affinity chromatography on glutathione-Sepharose beads, and analyzed tryptic digests by tandem mass spectrometry (MS/MS). Four peptides that cover the activation segment from L835 to R856, carrying one phosphoryl group at S841 (spectrum 1155) (data not shown), two phosphoryl groups at S841 and T844 (spectrum 1475) (data not shown), three phosphoryl groups at S840, S841, and S850 (spectrum 1976) (Fig. 2A), and three phosphoryl groups at S840, T844, and S850 (spectrum 2031) (Fig. 2B), were identified. In addition, a shorter peptide starting at T844 and carrying one phosphoryl group at S850 was identified (spectra 760 and 816) (data not shown). These autophosphorylation sites in the activation segment of WT Ire1 correspond to previously reported autophosphorylation sites in the activation segment (18).

We also characterized autophosphorylation of an L745A mutant of Ire1, which possesses an enlarged ATP binding pocket to accommodate chemically modified, bulkier adenine moieties (24, 25). *In vivo*, L745A-Ire1 supports induction of a UPRE-*lacZ* reporter to ∼60% of WT levels (23), which suggests that this mutant possesses significant protein kinase and RNase activities. MS/MS analyses of tryptic peptides derived from the L745A mutant identified several peptides that cover the activation segment from L835 to R856. One carried three phosphoryl groups at S840, S841, and T844 (spectrum 1583) (data not shown), another carried four phosphoryl groups at S840, S841, T844, and S850 (spectrum 2251) (data not shown), and a third one also carried four phosphoryl groups (spectrum 2263) (data not shown). Of these, three could be unambiguously assigned to S840, S841, and T844, while the forth phosphoryl group is located on S850 or T852. No peptide allowed unambiguous assignment of a phosphoryl group to T852. A peptide that covers the activation segment from K834 to R856 carried two phosphoryl groups at S837 and T844, one phosphoryl group at S840 or S841, and another phosphoryl group at S850, T852, or S853 (spectrum 1462) (Fig. 2C). Evidence for phosphorylation at S837 is provided by the *m*/*z* ratio of the ^+1^b ion of *m*/*z* 709.033 Da, which exceeds the *m*/*z* ratio for the unphosphorylated peptide by 79.7 Da. This difference matches, within the measurement error of 0.6 Da, the mass difference between a phosphorylated and unphosphorylated peptide. In addition to peptides carrying multiple phosphoryl groups, two monophosphorylated peptides, one covering the activation segment from L835 to R843 and being phosphorylated at S841 (spectrum 152) (data not shown) and one covering the activation segment from T844 to R856 and being phosphorylated at S850 (spectra 488 and 540) (data not shown), were detected. These data show that autophosphorylation of WT and L745A Ire1 in the activation segment strongly overlap and raise the possibility that S837 is an additional autophosphorylation site in the activation segment of Ire1.

### Mutation of all phosphorylation sites in the activation segment decreases, but does not eliminate, *HAC1* splicing, induction of ER chaperone genes, and survival of ER stress.

**FIG 3:**
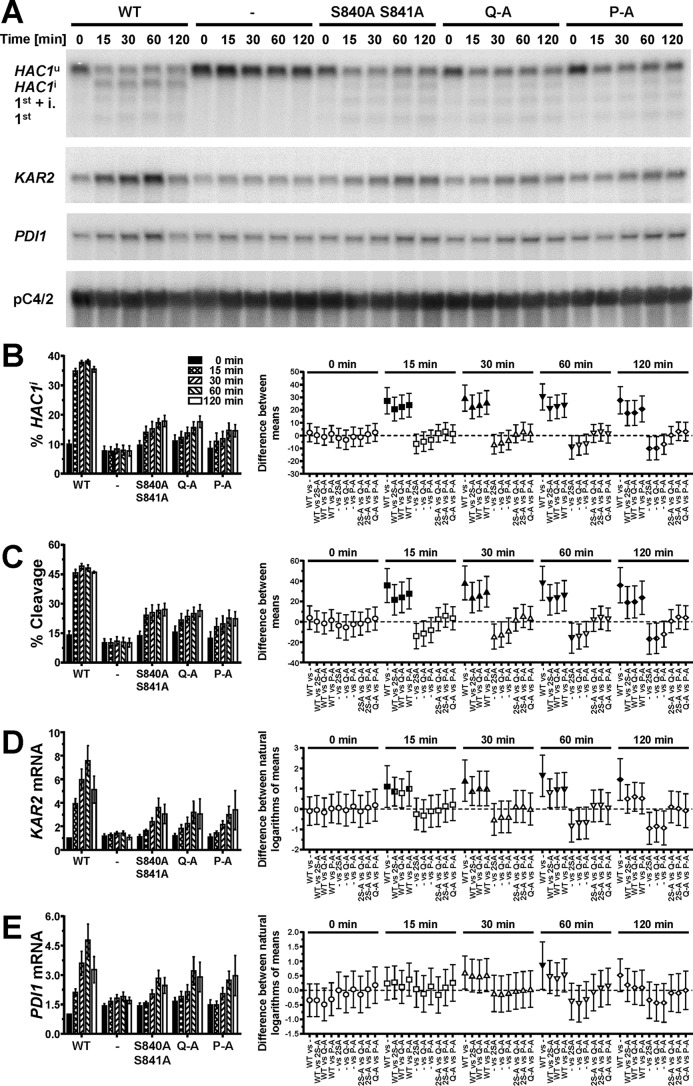
Mutation of phosphorylation sites in the activation loop decreases but does not abolish cleavage of *HAC1* mRNA by Ire1. (A) Northern blots for *HAC1*, *KAR2*, *PDI1*, and the loading control pC4/2 (53) on RNA extracted from *ire1*Δ strains expressing the indicated *IRE1* alleles from YCplac33 or carrying the empty vector (−). Mid-exponential-growth-phase cells were treated with 2 mM DTT for the indicated times. Abbreviations: *HAC1*^u^, unspliced *HAC1* mRNA; *HAC1*^i^, spliced *HAC1* mRNA; 1st + i., 1st exon of *HAC1*^u^ mRNA plus the intron; 1st, 1st exon of *HAC1*^u^ mRNA. (B to E) Quantification and 95% (open symbols), 99% (half-filled symbols), or 99.9% (filled symbols) confidence intervals of the percentage of *HAC1*^i^ mRNA (% *HAC1*^i^) (B), the percentage of *HAC1* mRNA cleavage (% Cleavage) (C), induction of *KAR2* (D), and induction of *PDI1* mRNAs (E). Bars represent the standard errors (*n* = 8 for the WT, *n* = 5 for all other strains). The confidence intervals for percent *HAC1*^i^, percent cleavage, and ln-transformed *KAR2* and *PDI1* mRNA levels were calculated using an ordinary two-way ANOVA with Tukey's correction for multiple comparisons (70). Abbreviations: 2S-A, S840A S841A; P-A, S837A S840A S841A T844A S850A; Q-A, S840A S841A T844A S850A.

To evaluate the contribution of S837 to activation of Ire1, we characterized the ER stress response of three Ire1 mutants, S840A S841A, S840A S841A T844A S850A (termed Q-A), and S837A S840A S841A T844A S850A (termed P-A). A time course experiment revealed that *HAC1* splicing reaches steady-state levels as early as 15 min after induction of ER stress with 2 mM dithiothreitol (DTT) in cells in the mid-exponential growth phase (Fig. 3A to C). An increase in *HAC1* splicing 15 min after induction of ER stress was detectable in all three mutants but was quantitatively lower than that in cells expressing WT Ire1. Differences between the three phosphorylation site mutants were not statistically significant (Fig. 3B and C). Two hours after induction of ER stress, the difference between cells lacking Ire1 and cells expressing either S840A-S841A-Ire1 or Q-A-Ire1 were statistically significant (Fig. 3B and C), whereas the difference between cells lacking Ire1 and cells expressing P-A-Ire1 did not become statistically significant (Fig. 3B and C). These two comparisons, cells expressing P-A-Ire1 to cells expressing S840A-S841A- and Q-A-Ire1 and cells expressing P-A-Ire1 to cells lacking Ire1, suggest that S837 makes a minor contribution to activation of Ire1.

The differences in *HAC1* splicing were reflected by the induction of *KAR2* and *PDI1* mRNAs. Induction of both mRNAs kinetically trailed *HAC1* splicing and reached a maximum only after ∼1 h of ER stress (Fig. 3A, D, and E). All three phosphorylation site mutants induced both *KAR2* and *PDI1* mRNAs to similar levels, which remained, especially in the case of *KAR2* mRNA, below the level of induction reached in cells expressing WT Ire1. Two hours after induction of ER stress with 2 mM DTT, *KAR2* mRNA levels, but not *PDI1* mRNA levels, were significantly increased in all autophosphorylation site mutants compared to cells lacking Ire1, suggesting that even P-A-Ire1 can partially activate expression of *KAR2* in ER-stressed cells.

**FIG 4:**
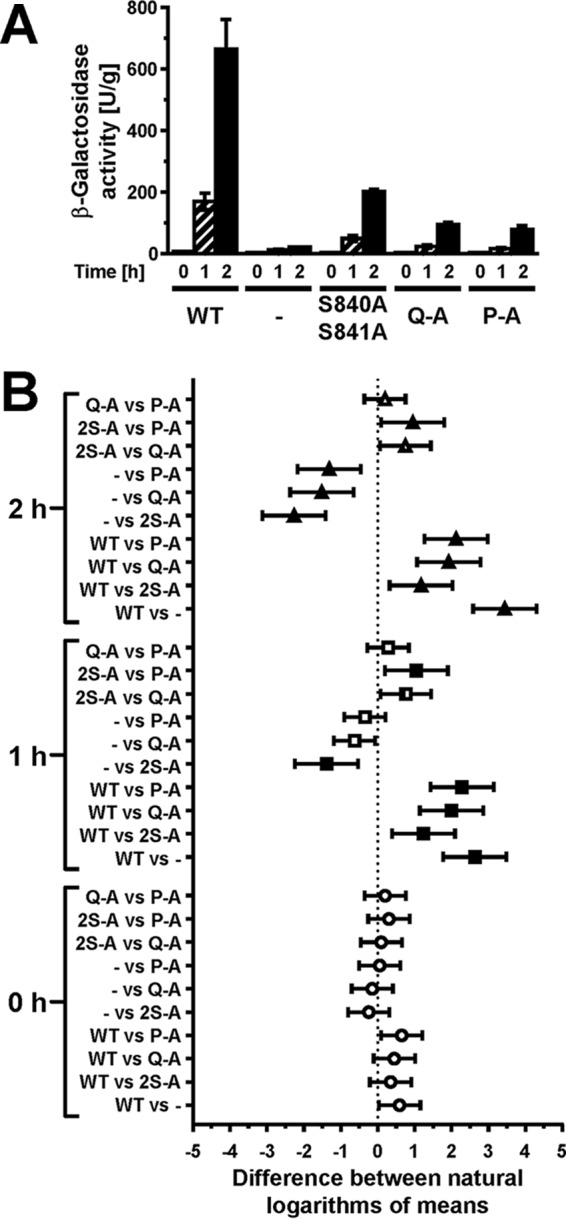
Mutation of all phosphorylation sites in the activation loop decreases, but does not abolish, induction of UPRE-*lacZ* reporters. (A) β-Galactosidase activity standardized to total cellular protein before and 1 h and 2 h after induction of ER stress with 2 mM DTT in mid-exponential *ire1*Δ cells expressing the indicated *IRE1* alleles from YCplac33 or carrying the empty vector (−). Bars represent standard errors (*n* = 3 for all strains). (B) 95% (open symbols), 99% (half-filled symbols), or 99.9% (filled symbols) confidence intervals (CI) were calculated for the ln-transformed data with an ordinary two-way ANOVA with Tukey's correction for multiple comparisons.

We next compared expression of a UPRE-*lacZ* reporter between cells expressing the different autophosphorylation site mutants, because accumulation of the comparatively stable protein β-galactosidase (26) may reveal subtle differences in transduction of the ER stress signal. All three autophosphorylation site mutants induced expression of the reporter, but they did so to a lower degree than cells expressing WT Ire1 (Fig. 4). This observation suggests that Ire1 can transduce an ER stress signal in the absence of autophosphorylation in its activation loop. Induction of the *lacZ* reporter was stronger in cells expressing S840A-S841A-Ire1 than in cells expressing either Q-A- or P-A-Ire1 (Fig. 4A), which supports the conclusion that phosphorylation of S837, T844, or S850 contributes to activation of Ire1, at least when phosphorylation at S840 and S841 is no longer possible.

**FIG 5:**
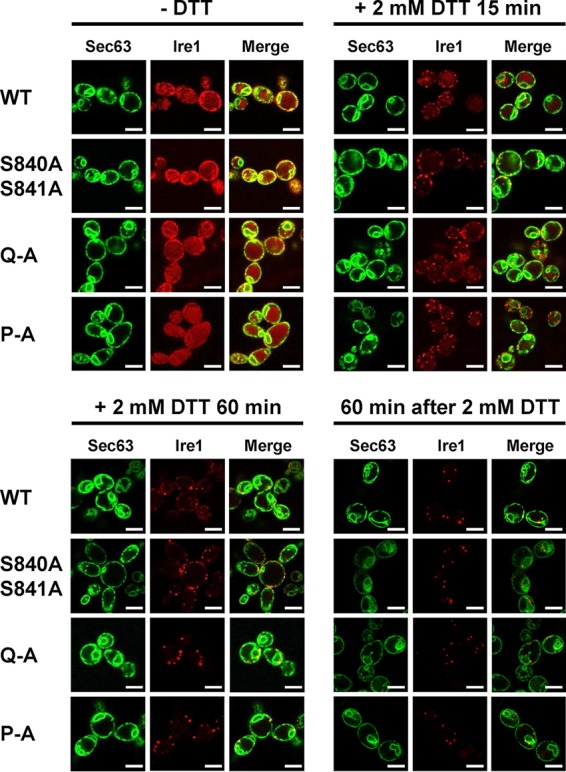
Activation loop phosphorylation is dispensable for clustering of Ire1 *in vivo*. Location of Sec63-GFP and Ire1-mCherry in unstressed cells and cells exposed to 2 mM DTT for 15 min or 1 h or after wash out of DTT for 1 h from cells treated with 2 mM DTT for 2 h. Sec63-GFP was expressed from the single-copy *URA3* plasmid pJK59 in *ire1*Δ cells transformed with single-copy *LEU2* plasmids derived from pEvA97 that carry the indicated *IRE1* alleles. Images covering ∼100 cells were taken, except for the DTT washout experiment, in which ∼20 cells were analyzed. Representative images are shown. Scale bars, 5 μm.

To explore the possibility that decreased *HAC1* splicing and *KAR2* and *PDI1* mRNA induction by activation loop mutants are caused by a defect in clustering of Ire1 *in vivo* (5), we monitored focus formation by WT and mutant Ire1 fused to the fluorescent protein mCherry before and after induction of ER stress with 2 mM DTT for 15 min or 60 min. Before induction of ER stress, Ire1-mCherry displayed a distribution characteristic for an ER protein with areas of fluorescence around the nucleus and the cell surface, which are indicative of the perinuclear and cortical ER (Fig. 5). This distribution also overlapped the distribution of a fluorescent marker for the ER, a fusion of green fluorescent protein (GFP) to the C terminus of the Sec63 subunit of the protein translocation channel of the ER membrane (27). In addition to this ER localization, cells carrying WT and mutant *IRE1* alleles also showed a distinct intracellular mCherry fluorescence that filled most of the cell body (Fig. 5). This mCherry fluorescence colocalizes with the fluorescence of the vacuolar stain 7-amino-4-chloromethylcoumarin (data not shown). As early as 15 min after induction of ER stress with 2 mM DTT, a characteristic punctate pattern developed for mCherry but not GFP fluorescence in cells expressing WT Ire1-mCherry. This punctate fluorescence is indicative of clustering of Ire1 *in vivo* in the ER membrane (5). One hour after DTT treatment, most of the mCherry fluorescence localizes to clusters (Fig. 5). There were no noticeable differences in the development of punctate mCherry fluorescence in cells expressing activation loop mutants of Ire1 fused to mCherry (Fig. 5). These experiments suggest that activation loop mutants do not display a defect in clustering *in vivo*.

**FIG 6:**
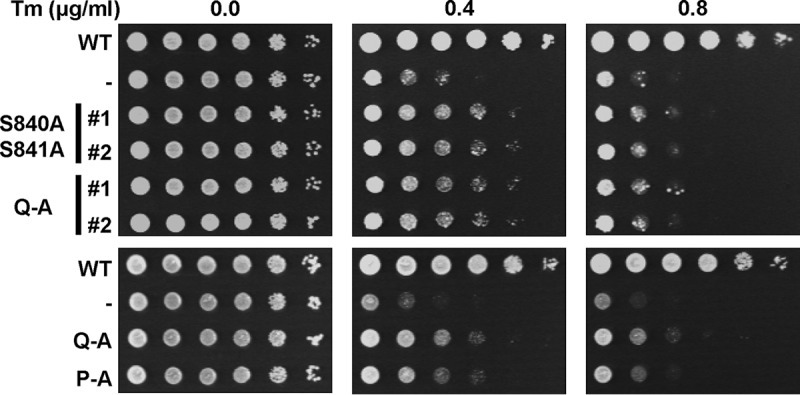
Survival of ER stress by activation loop mutants. Survival of ER stress induced with 0.4 and 0.8 μg/ml tunicamycin (Tm). Serial 10-fold dilutions of fresh overnight cultures of *ire1*Δ cells expressing the indicated *IRE1* alleles from YCplac33 or carrying the empty vector (−) were spotted on SD minus uracil plates containing 0.4 or 0.8 μg/ml Tm and allowed to grow for 2 to 3 days before taking photographs. Rows labeled #1 and #2 indicate two independent transformants for the S840A S841A and Q-A mutants. The experiment was repeated three times with qualitatively similar results.

**FIG 7:**
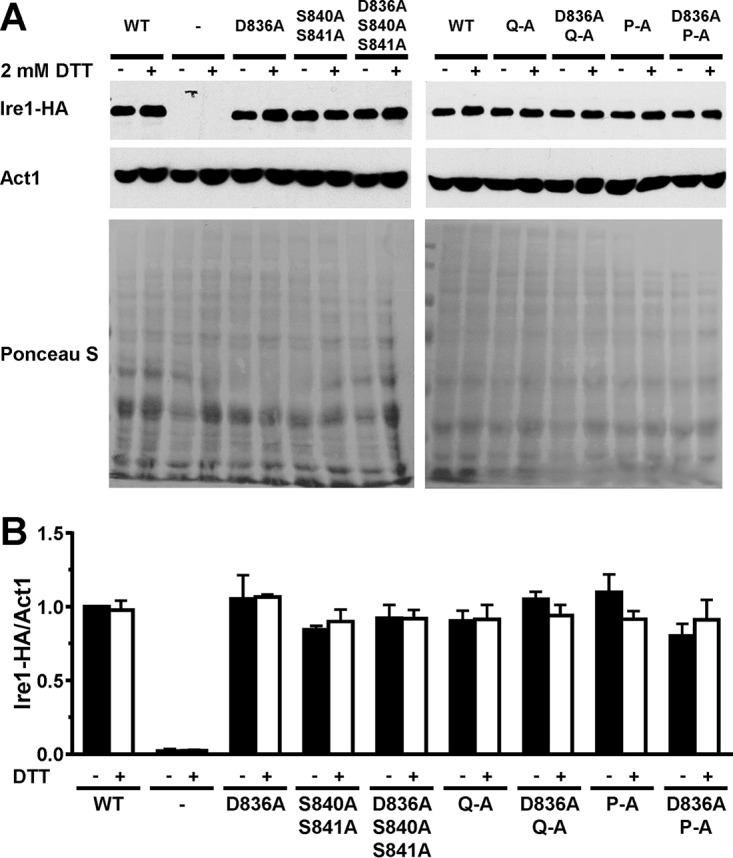
Expression of WT and mutant Ire1 proteins. (A) Western blots for HA-tagged Ire1 and Act1 isolated from mid-exponential-growth-phase *ire1*Δ strains expressing the indicated *IRE1* alleles from YCplac33 or carrying empty vector (−). Cells were treated for 2 h with 2 mM DTT where indicated (+). PVDF membranes were stained with Ponceau S after electrotransfer of proteins from 8% SDS-PAGE gels. (B) Quantification of Ire1-HA levels relative to the Act1 loading control. The expression level of Ire1-HA in untreated WT cells was arbitrarily set to 1.0. The data were analyzed with an ordinary two-way ANOVA with Tukey's correction for multiple comparisons. No significant differences in Ire1-HA expression levels were detected, except for the negative-control strain transformed with the empty vector. Bars represent standard errors (*n* = 7 for the WT; *n* = 4 for the Q-A, D836A Q-A, P-A, and D836A P-A mutants; and *n* = 3 for the D836A, S840A S841A, and D836A S840A S841A mutants and the strain transformed with empty vector).

To evaluate whether the weak ER stress response of the phosphorylation site mutants suffices to survive ER stress, we characterized the survival of these mutants under conditions of low levels of ER stress. Consistent with the gene expression data, all phosphorylation site mutants displayed a small degree of protection against ER stress, as evidenced by their improved growth on plates containing 0.4 μg/ml tunicamycin (Tm) compared to cells lacking Ire1 (Fig. 6). At higher concentrations of tunicamycin, this growth advantage of the phosphorylation site mutants over the *IRE1* deletion strain was diminished. Activation loop mutants express to levels comparable to those of WT Ire1 in both the S288C and W303 genetic backgrounds (Fig. 7 and data not shown), which suggests that the partial defect in responding to ER stress displayed by activation loop mutants cannot be explained by decreased intracellular abundance of mutant Ire1 proteins. In summary, these data show that all three autophosphorylation site mutants can activate a weak, but physiologically significant, ER stress response.

### D836 is required for the ER stress response mediated by phosphorylation site mutants.

The activation loop of Ire1 features an aspartate that has been functionally conserved throughout evolution in fungal and plant Ire1 (Fig. 1). Phosphorylation-independent RD kinases can employ a negatively charged glutamate located in the activation loop to stabilize the basic pocket formed by the invariant arginine and the basic amino acid located in strand β9 (20). For example, in phosphorylase kinase, glutamate 182 neutralizes the invariant arginine that precedes the catalytic aspartate (28). Mutation of this glutamate to serine decreases catalytic efficiency ∼20-fold (29). In partially active human cyclin-dependent kinase (CDK)–cyclin A complexes the basic pocket is stabilized by interaction with glutamate 162 (30). For these reasons, we introduced a D836A mutation into WT Ire1 and the three phosphorylation site mutants and compared the ER stress response of these mutants to the ER stress response of WT Ire1, S840A-S841A-Ire1, Q-A-Ire1, and P-A-Ire1.

**FIG 8:**
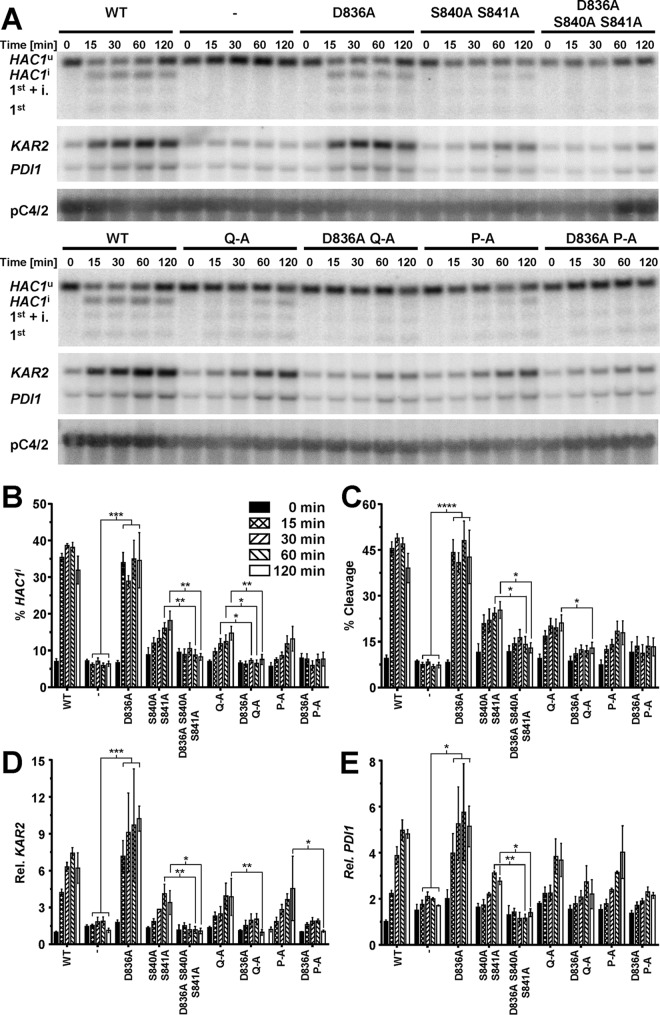
D836 is required for cleavage of *HAC1* mRNA by activation loop mutants. (A) Northern blots for *HAC1*, *KAR2*, *PDI1*, and the loading control pC4/2 (53) on RNA extracted from *ire1*Δ strains expressing the indicated *IRE1* alleles from YCplac33 or carrying the empty vector (−). Mid-exponential-growth-phase cells were treated with 2 mM DTT for the indicated times. (B to E) Quantification of the percentage of *HAC1*^i^ mRNA (% *HAC1*^i^) (B), the percentage of *HAC1* mRNA cleavage (% Cleavage) (C), induction of *KAR2* mRNA (D), and induction of *PDI1* mRNA (E). Bars represent standard errors. *, *P* ≤ 0.05; **, *P* ≤ 0.01; ***, *P* ≤ 0.001; ****, *P* ≤ 0.0001. *P* values for percent *HAC1*^i^ and percent cleavage were determined by Welch's test followed by a Games-Howell *post hoc* test (*n* = 12 for the WT, *n* = 4 for the empty vector transformed *ire1*Δ strain, and *n* = 6 for all other strains). *P* values for *KAR2* and *PDI1* induction were obtained from an ordinary two-way ANOVA with Tukey's correction for multiple comparisons on the ln-transformed data.

Splicing of *HAC1* mRNA and induction of both *KAR2* and *PDI1* mRNA were monitored in time course experiments similar to the experiments described above. Introduction of the D836A mutation into WT Ire1 had no effect on *HAC1* splicing or induction of *KAR2* and *PDI1* mRNAs (Fig. 8). In contrast, introduction of the D836A mutation into S840A-S841A-Ire1, Q-A-Ire1, or P-A-Ire1 resulted in a virtually complete loss of *HAC1*^i^ mRNA and abrogated induction of *KAR2* mRNA (Fig. 8). *PDI1* mRNA no longer increased to levels significantly above levels seen in cells deleted for *IRE1*. Despite the absence of *HAC1*^i^ mRNA, faint bands representing cleavage intermediates, such as the 1st exon of *HAC1*^u^ mRNA plus the intron and the 1st exon of *HAC1*^u^ mRNA, could still be observed in cells expressing D836A-S840A-S841A-, D836A-Q-A-, or D836A-P-A-Ire1 (Fig. 8A).

**FIG 9:**
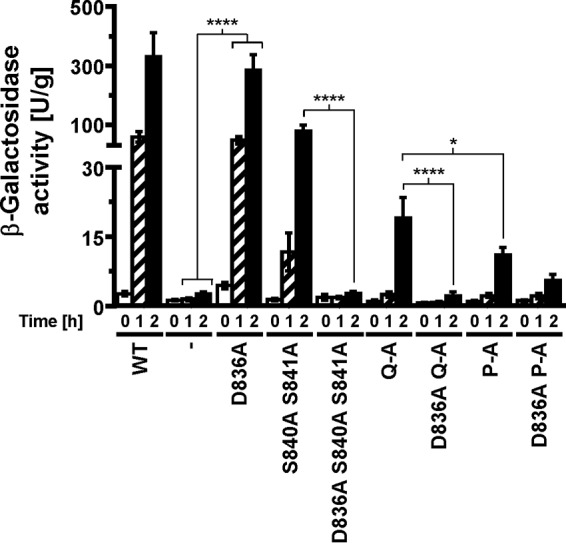
D836 is required for induction of UPRE-*lacZ* reporters by activation loop mutants. β-Galactosidase activity standardized to total cellular protein before and 1 h and 2 h after induction of ER stress with 2 mM DTT in mid-exponential *ire1*Δ cells expressing the indicated *IRE1* alleles from YCplac33 or carrying the empty vector (−). Bars represent standard errors (*n* = 12 for WT Ire1 and cells transformed with empty vector, *n* = 9 for all other strains). *, *P* ≤ 0.05; ****, *P* ≤ 0.0001. *P* values were obtained from an ordinary two-way ANOVA with Tukey's correction for multiple comparisons on the ln-transformed data.

**FIG 10:**
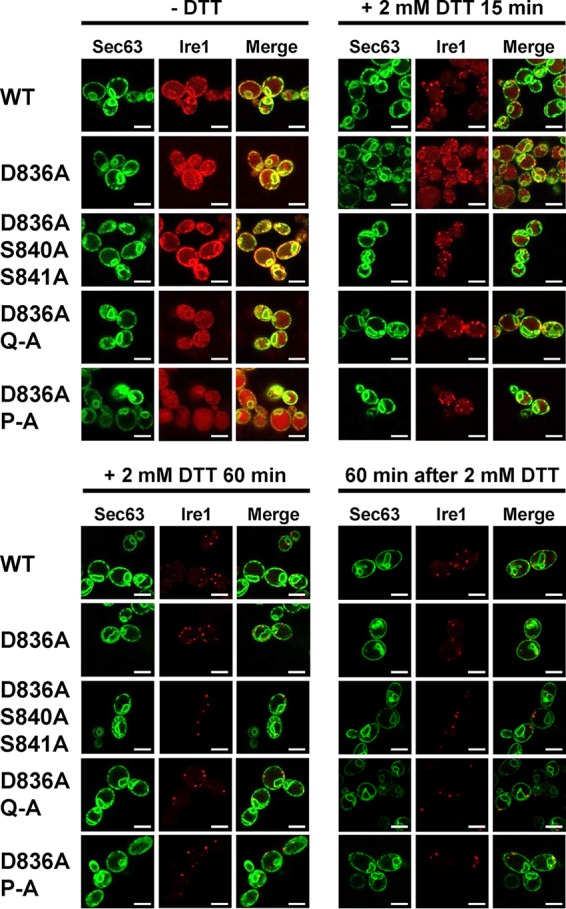
D836 is not required for clustering of Ire1 *in vivo*. Location of Sec63-GFP and Ire1-mCherry in unstressed cells and cells exposed to 2 mM DTT for 15 min or 1 h or after washout of DTT for 1 h from cells treated with 2 mM DTT for 2 h. Sec63-GFP was expressed from plasmid pJK59 in *ire1*Δ cells transformed with single-copy *LEU2* plasmids derived from pEvA97 that carry the indicated *IRE1* alleles. Images covering ∼100 cells were taken, except for the DTT washout experiment, in which ∼20 cells were analyzed. Representative images are shown. Scale bars, 5 μm.

**FIG 11:**
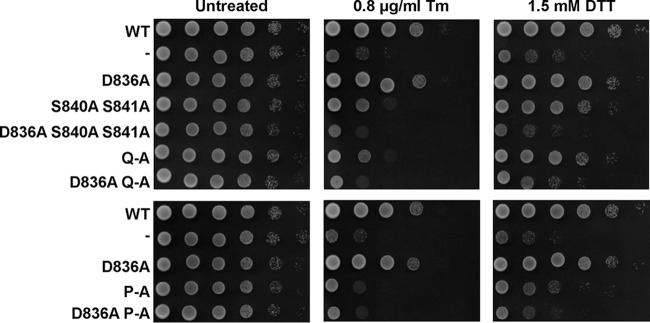
Survival of ER stress by activation loop mutants requires D836. Survival of ER stress induced with 0.8 μg/ml Tm or 1.5 mM DTT. Serial 10-fold dilutions of fresh overnight cultures of *ire1*Δ cells expressing the indicated *IRE1* alleles from YCplac33 or carrying the empty vector were spotted on SD-minus-uracil plates containing 0.8 μg/ml Tm or 1.5 mM DTT and allowed to grow for 2 to 3 days before taking photographs. The experiment was repeated three times with qualitatively similar results.

Introduction of the D836A mutation nearly completely eliminated expression of β-galactosidase from UPRE-*lacZ* reporters when introduced into S840A-S841A- and Q-A-Ire1 (Fig. 9). Introduction of the D836A mutation into P-A-Ire1 further decreased expression of β-galactosidase 2 h after induction of ER stress with 2 mM DTT (from 11.2 ± 1.6 U/g to 5.6 ± 1.4 U/g) to levels very close to and statistically undistinguishable from levels seen in *IRE1* deletion strains exposed to ER stress for 2 h (2.7 ± 0.4 U/g), but this decrease did not reach statistical significance. Differences in expression levels of the β-galactosidase reporter in *IRE1* deletion cells or cells expressing D836A-S840A-S841A-, D836A-Q-A-, or D836A-P-A-Ire1 were not statistically significant (Fig. 7). The D836A mutation, however, did not affect expression of β-galactosidase when introduced into WT Ire1 (Fig. 9). The D836A mutation also did not affect clustering of Ire1 *in vivo*, either in the context of otherwise WT Ire1 or the activation loop mutants (Fig. 10) or steady-state expression levels of Ire1 (Fig. 7). Introduction of the D836A mutations into the phosphorylation site mutants resulted in growth phenotypes very similar to the growth phenotypes of *IRE1* deletion cells (Fig. 11), but, as in the case of the gene expression data, the D836A mutation by itself did not decrease survival of ER stress induced with tunicamycin or DTT (Fig. 11). Taken together, these data support the conclusion that D836 is required for the residual ER stress response only when activation loop phosphorylation is impaired.

### Activation loop mutants do not affect inactivation of Ire1.

**FIG 12:**
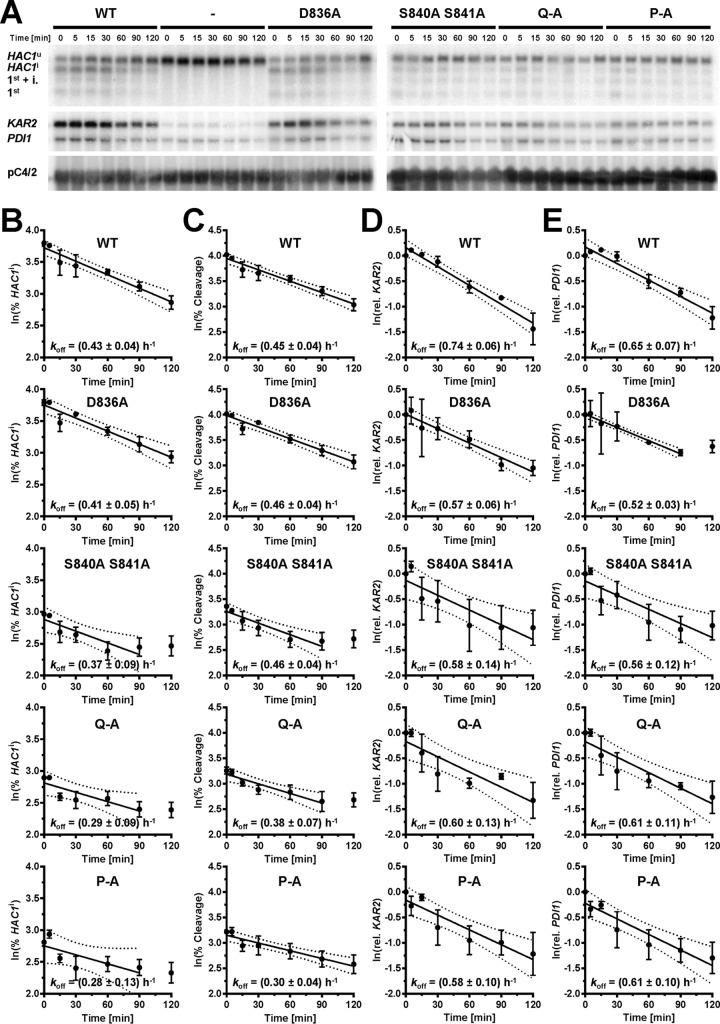
Mutation of phosphorylation sites in the activation loop does not alter inactivation of Ire1. (A) Northern blots for *HAC1*, *KAR2*, *PDI1*, and the loading control pC4/2 (53) on RNA extracted from *ire1*Δ strains expressing the indicated *IRE1* alleles from YCplac33 or carrying empty vector (−). Mid-exponential-growth-phase cells were treated with 2 mM DTT for 2 h before washing the cells once with culture medium and resuspending the cells in fresh, DTT-free medium. (B) Plot of the natural logarithm of the percentage of *HAC1*^i^ mRNA over time. (C) Plot of the natural logarithm of the percentage of *HAC1* mRNA splicing over time. (D) Plot of the natural logarithm of *KAR2* mRNA over time. (E) Plot of the natural logarithm of *PDI1* mRNA over time. Dotted lines represent the 95% confidence intervals of the linear regression models. The first-order rate constants, *k*_off_, were calculated from the slopes of the linear regression models.

To investigate whether mutations in the activation loop affect the inactivation of Ire1, we characterized the decay of *HAC1* splicing and *KAR2* and *PDI1* mRNA induction after washout of DTT from cells exposed to 2 mM DTT for 2 h. The percentage of *HAC1*^i^ mRNA, the percentage of *HAC1* mRNA cleavage, *KAR2* and *PDI1* mRNA levels decayed with first-order kinetics (Fig. 12). The lower maximal responses of the S840A S841A, Q-A, and P-A mutants to 2 mM DTT (Fig. 3, 8, and 12) allow these mutants to return to basal levels of *HAC1* splicing and *KAR2* and *PDI1* mRNA levels earlier than cells expressing the WT and D836A-Ire1 (Fig. 12). We did not find any statistically significant differences in the decay rates for any of the Ire1 mutants in an ordinary one-way analysis of variance (ANOVA) with Tukey's correction for multiple comparisons. Likewise, no obvious differences between WT Ire1 and any Ire1 mutants in the dissolution of Ire1 clusters at the ER membrane were observed (Fig. 5 and 10). These data suggest that inactivation of Ire1 is independent of D836 and phosphorylation sites in the activation loop.

### Negative regulation of Ire1 by the phosphatase Ptc2 requires phosphorylation sites in the activation loop of Ire1.

**FIG 13:**
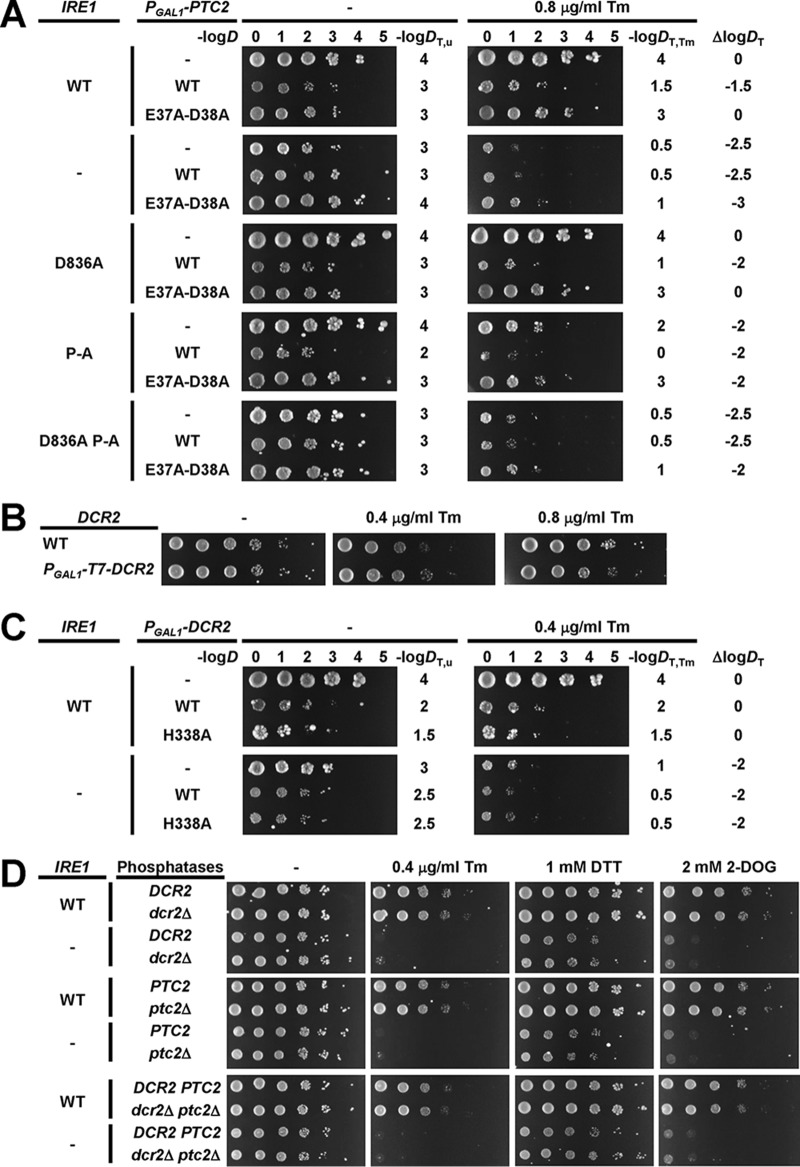
Mutation of all phosphorylation sites in the activation loop of Ire1 is epistatic to overexpression of Ptc2. (A) Effect of overexpression of WT and catalytically inactive E37A-D38A Ptc2 from the *GAL1* promoter on a 2μ plasmid on survival of ER stress induced with 0.8 μg/ml Tm. Fresh overnight cultures of *ire1*Δ cells expressing the indicated *IRE1* alleles from YCplac33 and the indicated *P_GAL1_-PTC2* alleles from pRSII422 were grown on 1% (wt/vol) raffinose and 2% (wt/vol) galactose and spotted in 10-fold serial dilutions onto plates containing 1% (wt/vol) raffinose and 2% (wt/vol) galactose and, where indicated, 0.8 μg/ml Tm. Plates were incubated for 7 days at 30°C. The negative decadic logarithms of the dilutions, *D*, of the 10-fold dilution series are shown on top of the plates. The threshold dilutions for untreated cells, *D*_T,u_, cells exposed to tunicamycin, *D*_T,Tm_, and the difference between both threshold dilutions, Δlog*D_T_*, are shown to the right of the plates. (B) Effect of overexpression of Dcr2 by placing the *GAL1* promoter in front of the endogenous *DCR2* gene on survival of ER stress. Serial 10-fold dilutions of fresh overnight cultures of *ire1*Δ cells and *ire1*Δ *P_GAL1_-T7-DCR2* cells expressing WT *IRE1* from YCplac33 grown on 1% (wt/vol) raffinose and 2% (wt/vol) galactose were spotted onto plates containing 1% (wt/vol) raffinose and 2% (wt/vol) galactose and, where indicated, 0.4 μg/ml or 0.8 μg/ml Tm. Plates were incubated for 4 days at 30°C. (C) Effect of overexpression of WT and catalytically inactive H338A Dcr2 from the *GAL1* promoter on a 2μ plasmid on survival of ER stress. Fresh overnight cultures of *ire1*Δ cells expressing the indicated *IRE1* alleles from YCplac33 and the indicated *P_GAL1_-DCR2* alleles from pRSII422 were grown on 1% (wt/vol) raffinose and 2% (wt/vol) galactose and spotted in 10-fold serial dilutions onto plates containing 1% (wt/vol) raffinose and 2% (wt/vol) galactose and, where indicated, 0.4 μg/ml Tm. Plates were incubated for 7 days at 30°C. (D) Deletion of both *DCR2* and *PTC2* does not affect survival of ER. Serial 10-fold dilutions of fresh overnight cultures of *ire1*Δ cells, *ire1*Δ *dcr2*Δ cells, *ire1*Δ *ptc2*Δ cells, and *ire1*Δ *dcr2*Δ *ptc*2Δ cells expressing the indicated *IRE1* alleles from YCplac33 were spotted onto SD-minus-uracil plates containing 0.4 μg/ml Tm, 1 mM DTT, or 2 mM 2-deoxy–d-glucose (2-DOG) to induce ER stress. Plates were incubated for 3 days at 30°C. All spotting assays were repeated at least once with qualitatively similar results.

The identical inactivation kinetics for WT Ire1 and activation loop mutants prompted us to characterize whether two negative regulators of Ire1, the phosphatases Dcr2 (31) and Ptc2 (32), negatively regulate Ire1 through its activation loop. Overexpression of Ptc2 from the *GAL1* promoter on a 2μ plasmid inhibited growth (32). Overexpression of WT Ptc2 but not catalytically inactive E37A-D38A- or D234A-Ptc2 also inhibited growth of cells exposed to a low concentration of tunicamycin (32). Consistent with this earlier report, we find that overexpression of Ptc2 from the *GAL1* promoter on the 2μ plasmid pRSII422 inhibited growth of unstressed cells (Fig. 13A). Overexpression of catalytically inactive E37A-D38A-Ptc2 also inhibited growth of unstressed cells, but it did so to a lesser extent than overexpression of WT Ptc2 (Fig. 13A). Deletion of *IRE1* slightly impaired growth of unstressed cells on raffinose and galactose but also masked the negative effects of expression of WT or E37A-D38A-Ptc2 on growth of unstressed cells (Fig. 13A). Likewise, expression of D836A-P-A-Ire1 masked the negative effects of expression of WT or E37A-D38A-Ptc2 on growth of unstressed cells, suggesting that WT and E37A-D38A-Ptc2 act through Ire1 to inhibit growth of unstressed cells.

These genetic interactions between *IRE1* and *PTC2* in unstressed cells complicate the interpretation of effects of overexpression of Ptc2 in ER-stressed cells expressing different *IRE1* alleles. Therefore, we semiquantitatively scored the effects of overexpression of Ptc2 on survival of ER stress by calculating the differences between the maximum dilutions at which growth of more than two cells can be observed for ER-stressed and unstressed cells. This scoring system revealed that expression of WT Ptc2 but not E37A-D38A-Ptc2 in cells expressing WT or D836A-Ire1 impaired growth in the presence of ER stress induced with 0.8 μg/ml tunicamycin (Fig. 13A). This observation is consistent with the earlier finding that overexpression of WT but not catalytically inactive Ptc2 inhibited growth of ER-stressed cells (32). The negative effects of overexpression of WT Ptc2 on growth of ER-stressed cells were abrogated in *ire1*Δ cells or cells expressing P-A- or D836A-P-A-Ire1 (Fig. 13A). These data show that deletion of *IRE1* or expression of a mutant in which all potential phosphorylation sites in the activation loop have been mutated to alanine mask the effects of overexpression of WT Ptc2 on growth of ER-stressed cells.

Overexpression of Dcr2 by placing the *GAL1* promoter in front of the chromosomal *DCR2* gene was reported to inhibit growth when ER stress was induced with tunicamycin (31). In contrast to this finding, we did not observe any inhibition of growth by overexpression of Dcr2 using a similar expression system (Fig. 13B). We then explored whether further elevation of Dcr2 levels by expressing Dcr2 from the *GAL1* promoter on the 2μ plasmid pRSII422 affects growth of ER-stressed cells, because overexpression of Ptc2 from the *GAL1* promoter on a 2μ plasmid inhibited growth of ER-stressed cells (Fig. 13A), while overexpression of Ptc2 by placing the *GAL1* promoter in front of the chromosomal *PTC2* gene had no effect on growth of ER-stressed cells (data not shown). Overexpression of WT Dcr2 and catalytically inactive H338A-Dcr2 (33) from the *GAL1* promoter on a 2μ plasmid inhibited growth of unstressed WT cells to the same extent but did not affect survival of ER stress (Fig. 13C). Deletion of *IRE1* slightly impaired growth on raffinose and galactose (Fig. 13C) and largely masked the negative effects of expression of WT and H338A-Dcr2 on growth of unstressed cells (Fig. 13C). Expression of WT or H338A-Dcr2 did not alter survival of ER stress by *ire1*Δ cells (Fig. 13C). These data suggest that overexpression of Dcr2 does not affect survival of ER stress.

To characterize whether deletion of *DCR2* or *PTC2* affects survival of ER stress, we constructed *dcr2*Δ and *ptc2*Δ strains and double *dcr2*Δ *ptc2*Δ strains. Survival of ER stress was not altered by deletion of *DCR2*, deletion of *PTC2*, or simultaneous deletion of both *DCR2* and *PTC2* (Fig. 13D). These data suggest that negative regulation of Ire1 by Dcr2 and Ptc2 to optimally tune the amplitude of the Ire1 signaling output is not required to survive ER stress or that other phosphatases exist that can compensate for the loss of both Dcr2 and Ptc2 in *dcr2*Δ *ptc2*Δ cells.

## DISCUSSION

The data presented here show that Ire1 can transduce a partial ER stress signal in the absence of phosphorylation in its activation loop (Fig. 3 and 4). The partial activation of *HAC1* splicing by activation loop mutants resulted in partial induction of *KAR2* and *PDI1* mRNAs (Fig. 3D and E) and partial protection from ER stress (Fig. 6). In addition, we show that transduction of this partial ER stress signal by activation loop mutants relies on the presence of a negatively charged amino acid, such as an aspartate or a glutamate, in the activation loop that has been conserved throughout evolution in fungal and plant Ire1 (Fig. 1). Mutation of this aspartate, D836 in S. cerevisiae Ire1, to alanine in activation loop mutants nearly completely eliminated production of *HAC1*^i^ mRNA, induction of *KAR2* and *PDI1* mRNA of a UPRE-*lacZ* reporter, and survival of ER stress (Fig. 8, 9, and 11). In this way, introduction of the D836A mutation into activation loop mutants is reminiscent of the *in vitro* behavior of human IRE1α. IRE1α lacks negatively charged amino acids in its activation loop (Fig. 1A) and displayed an ∼100-fold increase of its *V*_max_ and *k*_cat_ upon phosphorylation (22). In contrast, the D836A mutation by itself had no effect, because the levels of *HAC1* splicing, induction of *KAR2* and *PDI1* mRNA and of the UPRE-*lacZ* reporter, and survival of ER stress were indistinguishable from those of cells expressing WT Ire1. These observations suggest that D836 can partially substitute for activation loop phosphorylation in the activation loop mutants.

The role of activation loop phosphorylation in activation of Ire1 is thought to lie in conformational changes in the activation loop that result in opening of the ATP binding pocket (18). Binding of ATP then induces oligomerization of Ire1 and RNase activity (18). In this model for activation of Ire1, point mutations in the protein kinase domain, which largely inactivate the protein kinase activity of Ire1, should display a significant loss of RNase activity. However, mutation of D828, which contributes to coordinating two Mg^2+^ ions important for catalysis of the γ-phosphoryl transfer reaction, or mutation of the catalytic aspartate, D797, did not destroy RNase activity (7, 21). Retention of RNase activity by these protein kinase mutants may, as in the case for the activation loop mutants, be explained by the presence of D836 in the activation loop.

Based on its primary amino acid sequence, Ire1 belongs to the family of RD kinases, and its reliance on activation loop phosphorylation for full activity (Fig. 2 and 3) (22) suggests that Ire1 belongs to the family of phosphorylation-dependent RD protein kinases. In these protein kinases, phosphoamino acids in the activation loop move into a basic pocket formed by the invariant arginine that precedes the catalytic aspartate (R796 in S. cerevisiae Ire1) and a second basic amino acid located in strand β9 (K833 on strand β10 in Ire1). The crystal structure of oligomeric Ire1 shows phosphorylated T844 in contact with this basic pocket (4), as would be expected for a phosphorylation-dependent RD kinase. However, phosphorylation at T844 is of lesser importance than phosphorylation at S840 or S841, because induction of both *KAR2* and *PDI1* mRNAs was more severely affected in the S840A S841A double mutant than in the T844A single mutant (3). The importance of S840 or S841 in activation of Ire1 is further supported by the observation that all phosphorylation site mutants show a similar decrease in *HAC1* splicing and induction of both *KAR2* and *PDI1* (Fig. 3). This suggests that the other phosphorylation sites play only minor roles or require the presence of S840 or S841 to mediate activation of the endoribonuclease domain. Data obtained from the β-galactosidase reporter assays (Fig. 4) indicate that the other phosphorylation sites can mediate some activation of the endoribonuclease domain independent of S840 or S841. Introduction of the D836A mutation into any of the three phosphorylation site mutants nearly completely abolished *HAC1* splicing, induction of *KAR2*, *PDI1*, and the β-galactosidase reporter, and survival of ER stress (Fig. 8, 9, and 11). This behavior of the D836A mutation suggests that the residual ER stress response transduced by S840A-S841A-Ire1 relies on D836 and that D836 can partially substitute for functions provided by S840 or S841 in WT Ire1. In the absence of D836, S840, and S841, the other phosphorylation sites can no longer mediate effective activation of Ire1 (Fig. 8, 9, and 11), which suggests that a function provided by D836, S840, or S841 is necessary for activation of Ire1. At the same time, induction of the β-galactosidase reporter decreases from S840A-S841A-Ire1 to Q-A-Ire1 and P-A-Ire1 (Fig. 4), which suggests that the ability of D836 to mediate activation of Ire1 requires at least one of the other phosphorylation sites. These data are consistent with the view that the activation loop makes at least two contacts necessary for activation of Ire1, one mediated by S840 or S841 and in their absence and, to a lesser degree, by D836, and a second one mediated by one of the other phosphorylation sites.

The crystal structure of oligomeric Ire1 suggests that two contacts of the activation loop necessary for activation of Ire1 are made between phosphorylated S840 and S841 and R896 of the same molecule and K678 of an adjacent Ire1 molecule, as well as between phosphorylated T844 and the RD pocket (4). D836 may simply substitute for S840 and S841 by contacting the same basic pocket while phosphorylated T844 still remains in contact with the RD pocket, but it is also possible that D836 contacts the RD pocket and that phosphorylated T844, phosphorylated S837, or phosphorylated S850 contacts the basic pocket formed by R896 and K678 in S840A S841A mutants. Contacts made between the phosphorylated activation loop and basic pockets have been proposed to facilitate oligomerization of Ire1 (4). *In vivo* clustering of Ire1, however, was not affected by the activation loop mutants (Fig. 5) or by introduction of the D836A mutation into any of the activation loop mutants (Fig. 10). These observations suggest that surfaces other than the phosphorylated activation loop suffice to mediate efficient clustering of Ire1 *in vivo*. Therefore, it seems that the critical roles of activation loop phosphorylation in activation of Ire1 do not lie in facilitating clustering of Ire1.

D836 can only partially substitute for phosphorylation of S840 or S841, because all activation loop mutants can only support levels of *HAC1* splicing that are significantly lower than those in cells expressing WT Ire1. This may be due to the decreased negative charge of a carboxylate compared to a phosphate and subsequent partial neutralization of positive charges in basic pockets, such as the basic pocket formed by R896 and K678 of an adjacent Ire1 molecule, resulting in decreased stability of active conformations. In addition, steric constraints may prevent the smaller aspartate from moving as close to positive charges as phosphoamino acids might, which again may destabilize active conformations. Furthermore, D836 can only make one contact to basic pockets, whereas, for example, phosphorylated S840 and S841 can make two contacts to the same basic pocket (4). The lack of a phenotype for cells expressing D836A-Ire1 suggests that conformations formed by phosphorylated Ire1 are more stable or longer lived than any conformations in which D836 attempts to take over any roles of phosphorylation. Alternatively, phosphorylation may be kinetically outcompeting formation of any conformations in which D836 replaces phosphoserines or phosphothreonines.

Inactivation of Ire1 is thought to involve its dephosphorylation (21, 22) as well as its autophosphorylation in its αEF insertion loop (7, 21). The similar inactivation kinetics of WT Ire1 and activation loop mutants (Fig. 12) suggests that inactivation of Ire1 is a two-step process in which fast dephosphorylation of Ire1 precedes slower, phosphorylation-independent steps (Fig. 12). In the DTT washout experiments (Fig. 12), only the slower phosphorylation-independent steps may have been observed. These phosphorylation-independent steps may represent the reassociation of Kar2 with the luminal domain of Ire1 (34–37), the disassembly of Ire1 oligomers, or the clearance of misfolded and damaged proteins from the ER via ER-associated degradation or dilution by growth. We observed no noticeable differences in the disassembly of Ire1 foci in cells expressing WT Ire1, phosphorylation site mutants (Fig. 5), or a combination of phosphorylation site mutants and the D836A mutation (Fig. 10). Thus, while activation of Ire1 leading to *HAC1* splicing requires at least one negative charge in the activation loop, inactivation of Ire1 and dissolution of Ire1 clusters proceeds independent of negative charges on the activation loop.

The initial, fast dephosphorylation in the inactivation of Ire1 may be mediated by the phosphatases Dcr2 (31) and Ptc2 (32). Overexpression of Ptc2 in cells expressing different *IRE1* alleles revealed that deletion of *IRE1* or expression of *IRE1* alleles that lack all potential phosphorylation sites masks the effects of overexpression of Ptc2 (Fig. 13A). These findings are consistent with the view that Ptc2 attenuates Ire1 signaling by dephosphorylating the activation loop of Ire1, resulting in decreased levels of *HAC1* splicing (32). However, decreased phosphorylation of the activation loop in activation loop mutants may impair ATP binding (18) and decrease the protein kinase activity of Ire1, leading to decreased phosphorylation of other regions of Ire1, such as its αEF insertion loop. Therefore, the epistatic relationship between Ire1 and Ptc2 also may be explained by dephosphorylation of regions other than the activation loop by Ptc2. In contrast to Ptc2, we found no evidence that overexpression of Dcr2 affects survival of ER stress (Fig. 13B and C). We also do not observe a synthetic growth defect in *ire1*Δ *dcr2*Δ cells (Fig. 13D) or that deletion of *DCR2* impairs survival of ER stress, which contrasts with previously reported results (31). While these different results may be explained by the different genetic backgrounds, W303 and S288c, used in the two studies, we also did not observe any effects of overexpression of Dcr2 from the *GAL1* promoter on the 2μ plasmid pRS425 in the S288c genetic background (data not shown).

Deletion of *IRE1* or expression of D836A-P-A-Ire1 also masks the toxic effects of overexpression of WT and catalytically inactive Ptc2 and Dcr2 on growth of unstressed cells (Fig. 13A and C). For *PTC2*, epistatic relationships similar to those of *IRE1* seem to exist in unstressed and ER-stressed cells, suggesting that Ptc2 acts through the same mechanism in unstressed and ER-stressed cells. It is unlikely that unmasking of the inositol auxotrophy of *ire1*Δ cells (38) by overexpression of Ptc2 can explain the growth-inhibitory effects of Ptc2, because increasing the inositol concentration did not mitigate the growth-inhibitory effects of overexpression of Ptc2 (data not shown). Deletion of *IRE1* is also epistatic to overexpression of Dcr2 in unstressed cells (Fig. 13C). The lack of any effect of overexpression of *DCR2* on growth in the presence of ER stress suggests another genetic relationship between *IRE1* and *DCR2* besides that between *IRE1* and *PTC2*. Deletion of *IRE1* may, for example, perturb a secretory pathway function that is located upstream of a secretory pathway function of Dcr2.

In summary, our work shows that yeast Ire1 retains the ability to transduce a weak ER stress signal when all of its phosphorylation sites in its activation have been mutated to alanine. The ability of these activation loop mutants to respond to ER stress relies on the presence of a negatively charged amino acid in the activation loop. These findings provide a molecular explanation for some of the differences between yeast and mammalian Ire1.

## MATERIALS AND METHODS

### Plasmid constructions.

Plasmids were maintained in Escherichia coli XL10-Gold cells (no. 200314; Agilent Technologies, Stockport, United Kingdom). Standard protocols were used for plasmid constructions. The single-copy *URA*3 plasmid pJK59 (27), used for expression of Sec63-GFP, was obtained from W. A. Prinz (National Institute of Diabetes and Digestive and Kidney Diseases, National Institutes of Health, Bethesda, MD).

YCplac33-S840A-S841A-T844A-S850A-*IRE1*-HA (in brief, YCplac33-Q-A-*IRE1*-HA) was generated by cloning the 2,002-bp AfeI-MscI fragment of pC3060-S840A-S841A-T844A-S850A (18) into AfeI- and MscI-digested YCplac33-*IRE1*-HA (23). To generate YCplac33-S837A-S840A-S841A-T844A-S850A-*IRE1*-HA (in brief, YCplac33-P-A-*IRE1*-HA), the 310-bp PstI-SacI fragment of YCplac33-Q-A-*IRE1*-HA, was inserted into PstI- and SacI-digested pUC18 (39), and the S837A point mutation was introduced by QuikChange site-directed mutagenesis (Agilent Technologies) with primers H8293 and H8294 (Table 1). After confirmation of the mutagenized sequence by Sanger sequencing, the 3,103-bp PstI-SacI fragment was inserted into PstI- and SacI-digested YCplac33-Q-A-*IRE1*-HA (23). To introduce the D836A mutation into wild-type (WT) Ire1 and S840A-S841A-Ire1, the 1,405-bp HindIII-SacI fragments of YCplac33-*IRE1*-HA and YCplac-S840A-S841-*IRE1*-HA (23) were cloned into HindIII- and SacI-digested pUC18, followed by introduction of the D836A mutation by QuikChange site-directed mutagenesis with primers H8623 and H8624 in the case of WT Ire1 and primers H8625 and H8626 in the case of S840A-S841A-Ire1. After confirmation of the mutagenized sequence by Sanger sequencing, the 1,405-bp HindIII-SacI fragments of the two pUC18 plasmids were cloned into YCplac33-*IRE1*-HA to produce YCplac33-D836A-*IRE1*-HA and YCplac33-D836A-S840A-S841A-*IRE1*-HA, respectively. To introduce the D836A mutation into Q-A- and P-A-Ire1, the 1,578-bp BamHI-KpnI fragment of pGEX-1λT-Q-A-C′*IRE1* (S. Šestak and M. Schröder, unpublished data) was cloned into BamHI- and KpnI-digested pUC18, and the 69-bp EcoRV-PstI fragment was replaced with annealed and phosphorylated deoxyoligonucleotides H8627 and H8628 or annealed and phosphorylated deoxyoligonucleotides H8629 and H8630. The 1,578-bp KpnI-BamHI fragments of the resulting plasmids were cloned into BamHI- and KpnI-digested pAW42 (40). From the two resulting plasmids, pGEX-1λT-Q-A-C′*IRE1* and pGEX-1λT-P-A-C′*IRE1*, the 310-bp PstI-SacI fragments were cloned into PstI- and SacI-digested YCplac33-Q-A-*IRE1*-HA to generate YCplac33-D836A-Q-A-*IRE1*-HA and YCplac33-D836A-P-A-*IRE1*-HA.

**TABLE 1:** Oligodeoxynucleotides

Name	Sequence^*a*^
8691G	TGTGCAGGATCCCAAAGATTCAAAATTTTGCCGCC
DCR2-H338A-F	CAATGGTATGGGGAAAT**GC**CGACGACGAGGGAAGCT
DCR2-H338A-R	AGCTTCCCTCGTCGTCG**GC**ATTTCCCCATACCATTG
H4075A04	TGCCTTAGAACTTTCATAGC
H8293	GGTCTTTGCAAAAAACTAGAC**G**C**C**GG**C**CAGGCAGCATTTAGAGCAAAT
H8294	ATTTGCTCTAAATGCTGCCTG**G**CC**G**G**C**GTCTAGTTTTTTGCAAAGACC
H8623	TTGATATCAGACTTTGGTCTTTGCAAAAAACTAG**CTAGC**GGTCAGTCTTCATTTAGAACAAATTTGAATAACC
H8624	GGTTATTCAAATTTGTTCTAAATGAAGACTGACC**GCTAG**CTAGTTTTTTGCAAAGACCAAAGTCTGATATCAA
H8625	TTTGATATCAGACTTTGGTCTTTGCAAAAAACTAG**CTAGC**GGTCAGGCTGCATTTAGAACAAATTTG
H8626	CAAATTTGTTCTAAATGCAGCCTGACC**GCTAG**CTAGTTTTTTGCAAAGACCAAAGTCTGATATCAAA
H8627	ATCAGACTTTGGTCTTTGCAAAAAACTAG**CTAGC**GGTCAGGCAGCATTTAGAGCAAATTTGAATAACCCTGCA
H8628	GGGTTATTCAAATTTGCTCTAAATGCTGCCTGACC**GCTAG**CTAGTTTTTTGCAAAGACCAAAGTCTGAT
H8629	ATCAGACTTTGGTCTTTGCAAAAA**G**CTAG**C**CGCCGGCCAGGCAGCATTTAGAGCAAATTTGAATAACCCTGCA
H8630	GGGTTATTCAAATTTGCTCTAAATGCTGCCTGGCCGGCG**G**CTAG**C**TTTTTGCAAAGACCAAAGTCTGAT
H9327	TCATAAATACGGATACGTCTTTCTGTACCTCCATAGCCAGCATAACCACCAAGCTTCGTACGCTGCAGG
H9328	AGTTTTATACTTAAGTATCGAAGACCAGCACCGTGGTTAAAAATCTTAACAGGCCACTAGTGGATCTG
H9329	GCCGGAGGTCTTGCTCTTGGATTGGCTGGAAGGGTCAAGATTTTCTGCATAAGCTTCGTACGCTGCAGG
H9330	TCCCTAGGATTTTGACTATTCCATTGTTGTATAAAATATAGAGAACCAGAAGGCCACTAGTGGATCTG
H9331	ACTACCAAGTATAATAGGTACCTTTGATACAGCCTCGGTAACCGGATCATACCCATTTGCTGTCCACCAG
H9332	CCGGAGTGGCTCTCTTTATCAATTACCGGGTTTGATAGAATTTGTCCCATACCCATTTGCTGTCCACCAG
PTC2-E37A-D38A-F	ACATTAGGCTCTAGAATGTGTGAA**G**CC**G**CCAT**G**GACATCCGCCACC
PTC2-E37A-D38A-R	GGTGGCGGATGTC**C**ATGG**C**GG**C**TTCACACATTCTAGAGCCTAATGT
U5803H01	TAAATGGCTAGCATGACTGGTGGACAGCAAATGGGTG
U5803H02	GATCCACCCATTTGCTGTCCACCAGTCATGCTAGCCATTTAAT

a Restriction sites are underlined. Mutagenic base substitutions in oligodeoxynucleotides used for site-directed mutagenesis are shown in boldface.

For tagging Ire1 with mCherry, the 2,013-bp AfeI-SacI fragment of pEvA97 (5) was cloned into the corresponding AfeI- and SacI-digested YCplac33-*IRE1*-HA plasmids. The 3,356-bp HindIII-NheI fragments of the resulting YCplac33-*IRE1*-HA plasmids then were reinserted into pEvA97, because mCherry fluorescence was not detectable in unstressed cells when the mCherry-Ire1 fusion protein was expressed from YCplac33 in initial experiments. pGEX-1λT-L745A-C′IRE1 was constructed by amplifying a 1,277-bp PCR product with primers 8619G and H4075A04 from YCplac33-L745A-*IRE1* (23) and cloning the BamHI- and PmlI-digested PCR product into BamHI- and PmlI-digested pAW42.

For galactose-inducible expression of hemagglutinin (HA)- and protein A-tagged Dcr2 and Ptc2, the 2,777-bp and 2,435-bp BssHII fragments of BG1805-*DCR2* and BG1805-*PTC2* (41) were ligated to the 6,692-bp BssHII fragment of pRSII422 (42) or the 6,676-bp BssHII fragment of pRS425 (43). H338A-Dcr2 (31) and E37A-D38A-Ptc2 (32) were generated by QuikChange site-directed mutagenesis with primer pairs DCR2-H338A-F and DCR2-H338A-R as well as PTC2-E37A-D38A-F and PTC2-E37A-D38A-R (Table 1) on BG1805-*DCR2* and BG1805-*PTC2*, respectively. After confirmation of the mutagenized sequences by Sanger sequencing, the 1,070-bp ClaI-EagI fragment of BG1805-H338A-*DCR2* was cloned into ClaI- and EagI-digested pRSII422-*DCR2*, and the 1,153-bp SacI-XhoI fragment of BG1805-E37A-D38A-*PTC2* was cloned into SacI- and XhoI-digested pRSII422-*PTC2*. From there, the 2,777-bp BssHII fragments of pRSII422-*DCR2* and pRSII422-H338A-*DCR2* and the 2,435-bp BssHII fragments of pRSII422PTC2 and pRSII422-E37A-D38A-*PTC2* were cloned into BssHII-digested pRS425.

### Yeast methods.

Yeast strains (Table 2) were transformed by the lithium acetate (LiOAc) method (46). Ire1 was deleted in W303-1A (44) as described previously (47). *DCR*2 and *PTC2* were deleted by PCR-based gene disruption (48) with primer pairs H9327 and H9328 as well as H9329 and H9330 (Table 1), with pFA6a-*kan*MX2 (48) or pFA6a-*hph*NT1 (49) as the template. The *GAL1* promoter and an N-terminal T7 epitope tag were introduced by transforming PWY 260 with PCR constructs amplified with primer pairs H9328 and H9331 as well as H9330 and H9332 from plasmid pFA6a-*kan*MX6-*P_GAL1_*-T7. pFA6a-*kan*MX6-*P_GAL1_*-T7 was constructed by inserting annealed and kinased oligonucleotides U5803H01 and U5803H02 into PacI- and BamHI-digested pFA6a-*kan*MX6-*P_GAL1_* (50). The T7 sequence in pFA6a-*kan*MX6-*P_GAL1_*-T7 was confirmed by Sanger sequencing. In-frame fusion of the T7 epitope tag to the *DCR2* and *PTC2* open reading frames was confirmed by Sanger sequencing.

**TABLE 2:** Saccharomyces cerevisiae strains

Name	Genotype^*a*^	Reference
W303-1A		44
PWY 260	*ire1*Δ::*TRP1 his3-11*,*15*::*HIS3*^+^ UPRE-*lacZ leu2-3*,*112*::*LEU2*^+^ UPRE-*lacZ*	23
MSY 14-02	*ire1*Δ::*kanMX2*	This study
MSY 792-02	*ire1*Δ::*TRP1 dcr2*Δ::*kanMX2 his3-11*,*15*::*HIS3*^+^ UPRE-*lacZ leu2-3*,*112*::*LEU2*^+^ UPRE-*lacZ*	This study
MSY 793-06	*ire1*Δ::*TRP1 ptc2*Δ::*kanMX2 his3-11*,*15*::*HIS3*^+^ UPRE-*lacZ leu2-3*,*112*::*LEU2*^+^ UPRE-*lacZ*	This study
MSY 794-11	*ire1*Δ::*TRP1 kanMX6-P_GAL1_*_,_*_10_-T7-DCR2 his3-11,15*::*HIS3*^+^ UPRE-*lacZ leu2-3*,*112*::*LEU2*^+^ UPRE-*lacZ*	This study
MSY 795-01	*ire1*Δ::*TRP1 kanMX6-P_GAL1_*_,_*_10_-T7-PTC2 his3-11,15*::*HIS3*^+^ UPRE-*lacZ leu2-3*,*112*::*LEU2*^+^ UPRE-*lacZ*	This study
MSY 796-02	*ire1*Δ::*TRP1 dcr2*Δ::*kan*MX2 *ptc2*Δ::*hphNT1 his3-11*,*15*::*HIS3*^+^ UPRE-*lacZ leu2-3*,*112*::*LEU2*^+^ UPRE-*lacZ*	This study
MSY 797-02	*ire1*Δ::*TRP1 dcr2Δ*::*hphNT1 ptc2*Δ::*kanMX2 his3-11*,*15*::*HIS3*^+^ UPRE-*lacZ leu2-3*,*112*::*LEU2*^+^ UPRE-*lacZ*	This study
Y01907	*MAT***a** *his3*Δ*1 leu2*Δ*0 met15*Δ*0 ura3*Δ*0 ire1*Δ::*kanMX4*	45

a All strains, except Y01907, carry the alleles *MAT***a**, *ade2-1*, *can1-100*, *his3-11*,*15*, *leu2-3*,*112*, *trp1-1*, and *ura3-1*.

To induce ER stress, cells were grown to mid-exponential growth phase on synthetic dextrose (SD) medium lacking uracil or leucine (51). DTT was added to a final concentration of 2 mM from a 1 M stock solution made in water. To wash out DTT, cells treated with 2 mM DTT for 2 h were washed once with SD medium lacking uracil and then resuspended in the same culture volume of fresh SD medium lacking uracil. For fluorescence microscopy, the concentrations of adenine and l-tryptophan were raised to 100 mg/liter. Survival of ER stress was scored by spotting 2 μl of serial 10-fold dilutions of fresh overnight cultures onto freshly prepared synthetic medium agar plates lacking uracil and containing 2% (wt/vol) glucose as the carbon source. Expression of *DCR2* and *PTC2* from the *GAL1* promoter was induced by growing the cells from single colonies in a small volume of synthetic medium with 1% (wt/vol) raffinose and 2% (wt/vol) galactose as carbon sources lacking uracil, both uracil and adenine, or both uracil and leucine. Cells were then spotted onto synthetic medium plates containing 1% (wt/vol) raffinose and 2% (wt/vol) galactose for ER stress survival assays. All overnight cultures were adjusted to optical densities at 600 nm of 3.0 before preparing serial 10-fold dilutions. To semiquantitatively evaluate growth assays, threshold dilutions, *D_T_*, were defined as the maximum dilutions at which growth could be observed and were determined with an accuracy limit of ∼0.5. The difference between the two negative decadic logarithms of the threshold dilutions for tunicamycin-exposed cells and untreated cells, Δlog *D_T_*, is defined as Δlog *D_T_* = −log *D*_T,Tm_ − −log *D*_T,u_, where *D*_T,Tm_ is the threshold dilution of tunicamycin-exposed cells and *D*_T,u_ is the threshold dilution of untreated cells. Only differences in Δlog *D_T_* of ≥0.5 were considered significant.

### Northern analysis.

Northern blotting was performed as described previously (47), except that cells were lysed by bead beating with 0.5-mm-diameter glass beads (no. 11079105z; Stratech Scientific, Newmarket, United Kingdom) in a Precellys 24 instrument (Bertin Technologies, Montigny-le-Bretonneux, France) at 4°C with two cycles of 30 s at 6,500 rpm, separated by a 30-s break. The probes for *HAC1* (47), *KAR2* (52), *PDI1* (52), and the loading control, pC4/2 (53), were described previously. All mRNAs were quantified by phosphorimaging on a Typhoon 9400 system (GE Healthcare, Little Chalfont, United Kingdom). Volumetric measurements were normalized to the loading control, pC4/2. The percentage of *HAC1*^i^ is defined as 100 × *HAC1*^i^/(*HAC1*^u^ + *HAC1*^i^ + 1st exon and intron + 1st exon), and the percentage of *HAC1* cleavage is defined as 100 × [*HAC1*^i^ + 0.5 × (1st exon and intron + 1st exon)]/(*HAC1*^u^ + *HAC1*^i^ + 1st exon and intron + 1st exon).

### β-Galactosidase reporter assays.

β-Galactosidase reporter assays were performed as described before (52). Cells were grown to mid-exponential phase, a 0-h sample was collected, the remainder of the culture was exposed to 2 mM DTT, and further samples were collected after 1 h and 2 h. After washing cells with ice-cold water, protein extracts were produced in 1× reporter lysis buffer (no. E3971; Promega, Southampton, United Kingdom) by bead beating with 0.5-mm-diameter glass beads in a Precellys 24 instrument at 4°C with 3 cycles of 10 s at 6,500 rpm. Between each cycle, samples were cooled for 5 min on ice. The lysates were centrifuged at 12,000 × *g* and 4°C for 10 min, and supernatants were transferred into fresh microcentrifuge tubes to obtain protein extracts. β-Galactosidase activity was standardized to total protein determined with the DC protein assay from Bio-Rad Laboratories (no. 500-0116; Hemel Hempstead, United Kingdom).

### Protein extraction and Western blotting.

Mid-exponential-growth-phase cells were washed once with 5 ml of ice-cold water and once with 1 ml ice-cold water and then resuspended in 200 μl 8 M urea, 2.5% (wt/vol) SDS, 50 mM Tris-HCl, pH 7.5 (at 4°C), 6 mM EDTA, 5 mM β-mercaptoethanol, 2 mM phenylmethylsulfonyl fluoride (PMSF), and 6 mM 4-(2-aminoethyl)-benzenesulfonyl fluoride (AEBSF). The cells were lysed by bead beating as described for the β-galactosidase reporter assays. Protein concentrations were quantified with bicinchoninic acid (54) after alkylation of β-mercaptoethanol with 0.1 M iodoacetamide in 0.1 M Tris-HCl, pH 8.0, at 37°C for 15 min.

Proteins (50 μg) were separated on 8% SDS-PAGE gels (55) and transferred to polyvinylidene difluoride (PVDF) membranes (Amersham HyBond-P; no. RPN303F; pore size, 0.45 μm; GE Healthcare, Little Chalfont, United Kingdom) by semidry electrotransfer in 0.1 M Tris, 0.192 M glycine, and 5% (vol/vol) methanol at 2 mA/cm^2^ for 75 min. Membranes were stained with 0.5% (wt/vol) Ponceau S in 1% (vol/vol) acetic acid for 5 min at room temperature (RT), destained three times for 2 min each with water, air dried, and photographed. Membranes were then blocked overnight in 5% (wt/vol) skim milk powder in TBST (20 mM Tris-HCl, pH 7.6 [at RT], 137 mM NaCl, and 0.1% [vol/vol] Tween 20) at 4°C and then incubated with a 1:1,000 dilution of rabbit anti-HA antibody (no. H6908; batch no. 015M4868V; Sigma-Aldrich, Poole, United Kingdom) in TBST plus 5% (wt/vol) skim milk powder for 2 h at RT. Blots were washed four times with TBST and then probed with a 1:2,000 dilution of anti-rabbit IgG (H+L)-peroxidase conjugate (no. 7074S; batch no. 24; Cell Signaling Technology Inc., Danvers, MA, USA) in TBST plus 5% (wt/vol) skim milk powder for 1 h at RT. Blots were then washed four times with TBST and developed by enhanced chemiluminescence as described before (56). To probe blots for the actin loading control, blots were stripped after enhanced chemiluminescence detection by washing them twice for 5 min with 100 mM Tris-HCl, pH 8.5 (at RT), and 0.1% (vol/vol) Tween 20; twice for 15 min with 100 mM Tris-HCl, pH 8.5 (at RT), 200 mM β-mercaptoethanol, and 0.1% (vol/vol) Tween 20; twice for 5 min with TBST; twice for 15 min with 100 mM glycine, pH 2.5 (at RT), and 0.1% (wt/vol) Tween 20 at 37°C; and twice for 15 min with TBST. Blots were then blocked overnight at 4°C with 5% (wt/vol) skim milk powder in TBST and then incubated for 1 h at RT with a 1:1 × 10^5^ dilution of a mouse anti-β-actin antibody (no. ab170825; batch no. GR184354-8; Abcam, Cambridge, United Kingdom) in TBST plus 5% (wt/vol) skim milk powder. Blots were washed four times with TBST, incubated for 1 h at RT with a 1:2 × 10^5^ dilution of a goat anti-mouse IgG (H+L) peroxidase conjugate (no. 31432; batch no. OE17149612; Thermo Fisher Scientific, Loughborough, United Kingdom) in TBST plus 5% (wt/vol) skimmed milk powder, washed again four times with TBST, and developed by enhanced chemiluminescence. Films were scanned on an MFC-5320DW all-in-one printer (Brother Industries, Manchester, United Kingdom) and saved as tif files. Bands were quantified using CLIQS 1.1 (Totallab, Newcastle upon Tyne, United Kingdom).

### Protein expression and purification.

The cytosolic domains of WT and L745A-Ire1 starting at amino acid 556 were expressed as N-terminal glutathione *S*-transferase (GST) fusion proteins from plasmid pGEX-1λT (GenBank accession no. U13849) by autoinduction (57) in E. coli BL21-CodonPlus(DE3)-RIL cells (no. 230240; Agilent Technologies). These constructs encompass the complete linker, serine/threonine protein kinase, and RNase domains of Ire1. A ZYM-5052 culture containing 50 μg/ml ampicillin and 25 μg/ml chloramphenicol was inoculated with a 1/1,000 volume of a fresh overnight culture grown in MDG medium, grown at 37°C for 5 h, and then grown for a further 28 h at 20°C (57). The cells were harvested by centrifugation at 4,750 × *g* and 4°C for 15 min and washed twice with ice-cold 0.2 M Tris-HCl, pH 8.0 (at 4°C), and finally resuspended in 0.2 M Tris-HCl, pH 8.0 (at 4°C), 0.5 M sucrose, one tablet of Roche complete protease inhibitors lacking EDTA (no. 11873580001; Roche Applied Sciences, Burgess Hill, United Kingdom), 2 mM PMSF, 6 mM AEBSF, and 1 μg/ml pepstatin. The cells were lysed by addition of 10 μg/OD_600_ chicken lysozyme (no. 62971; Sigma-Aldrich), addition of EDTA to a final concentration of 1 mM, dilution of the cell suspension with one volume of H_2_O, and incubation of the cell suspension on a roller mixer for 10 min at room temperature. After addition of a 1/10 volume of 10% (vol/vol) Triton X-100, the cell suspension was sonicated in a Soniprep 150 sonicator (MSE Ltd., London, United Kingdom) with a 19-mm-diameter probe in an ice-NaCl bath for 12 cycles of 1-min sonications with an amplitude of 0.22 μm followed by a cooling period in which the cell suspension was allowed to cool to 4°C. The lysate was then cleared by centrifugation at 40,000 × *g* and 4°C for 15 min. The GST-Ire1 fusion protein was purified by affinity chromatography on GSTrap 4B Sepharose columns (no. 28-4017-45; GE Healthcare) and eluted from the column with 20 mM Tris-HCl, pH 7.5 (at 4°C), 100 mM NaCl, 5% (vol/vol) glycerol, 0.1% (wt/vol) 3-[(3-cholamidopropyl)-dimethylammonio]-1-propanesulfonate (CHAPS), 1 mM EDTA, 2 mM PMSF, and 10 mM glutathione. The eluted protein was concentrated in Amicon Ultra-15 centrifugal filters (molecular size cutoff of 50 kDa; no. UFC905008; Merck Millipore) and desalted on a HiTrap desalting column (no. 17-1408-01; GE Healthcare).

### MS identification of Ire1 autophosphorylation sites.

Five micrograms of the cytosolic domains of WT and L745A-Ire1, expressed as GST fusion proteins in E. coli and purified by affinity chromatography on GSTrap 4B columns as described above, were run on an 8% SDS-PAGE gel and stained with Coomassie brilliant blue R250. Coomassie-stained protein bands were excised from the gel, transferred to a microtiter plate, and then destained, buffer exchanged, reduced with DTT, alkylated with iodoacetamide, and digested with trypsin using an automated digestion robot (DiGilab, Genomic Solutions, Ann Arbor, MI) as described before (58).

MS/MS analysis of the cytosolic domains of WT and mutant Ire1 expressed in E. coli was carried out on an LTQ XL Orbitrap mass spectrometer (Thermo Scientific) coupled to an Ultimate 3000 nano-high-performance liquid chromatography (HPLC) system. The reverse-phase liquid chromatography system consisted of a desalting column (300 μm by 5 mm; 3 μm; 100 Å; PepMap C_18_) and an analytical column (75 μm by 250 mm; 3 μm; 100 Å; PepMap C_18_) with split solvent delivery (split ratio of 1:300). A Thermo nanospray II source was fitted with a 30-μm silica emitter tip (PicoTip; New Objective) and maintained at a 1,100-V ion spray voltage. Peptide samples (3 μl) were loaded onto the trap column in 0.1% (wt/vol) trifluoroacetic acid at 20 μl/min for 3 min and eluted at 300 nl/min using a gradient from 0.05% (vol/vol) formic acid in water (buffer A) to 0.05% formic acid in 80% (vol/vol) acetonitrile (buffer B). The gradient profile was 4% buffer B for 3 min, 4% B to 40% B over the course of 40 min, 40% B to 65% B over the course of 6 min, 65% B to 95% B over the course of 1 min, and 95% B for 6 min. Using Excalibur 2.0.1, intact peptides were detected between *m/z* 400 and *m/z* 1,600 in the Orbitrap at a resolution of 60,000. Internal calibration was performed using the ion signal of (Si(CH_3_)_3_O)_6_H^+^ at *m/z* 445.120025 as a lock mass (59). Maximum ion accumulation time allowed on the LTQ Orbitrap was 1 s for all scan modes. Automatic gain control was used to prevent overfilling of the ion trap. Collision-induced dissociation (CID) spectra of the top 5 peptide ions (minimum intensity of 10,000 counts, rejection of singly charged precursors) were acquired at a normalized collision energy of 35. Dynamic exclusion was set with a repeat count of 1, a repeat time of 30 s, an exclusion time of 3 min, and an exclusion list size of 50. The chromatography feature was enabled with a correlation area ratio of 1.0. Activation Q was set to 0.25 with a 30-ms activation time.

XITandem tornado version 2009.04.01.1 (60) was used to search the ENSEMBL yeast proteome database (version SGD1.01.55) and the common repository of adventitious proteins (cRAP; http://www.thegpm.org/crap/index.html) for the identification of proteins and the reversed version of both databases. Precursor mass accuracy was set to 20 ppm and fragment mass error to 0.6 Da. Carbamidomethylation of cysteine was set as a fixed modification and phosphorylation of serine, threonine, and tyrosine as a variable modification. At the refinement step, oxidation of methionine and tryptophan, double oxidation of methionine and tryptophan, deamidation of asparagine and glutamine, methylation of aspartate, glutamate, histidine, lysine, arginine and asparagine, carbamidomethylation of lysine, histidine, aspartate, and glutamate, and dehydration of serine and threonine were included in the search parameters. At a cutoff log_*e*_ value of 1.0, searching the decoy databases indicated a false discovery rate between 1.18% and 1.39% for protein identification. Detailed results and all spectra can be accessed via the following links: WT, http://human.thegpm.org/thegpm-cgi/plist.pl?path=/tandem/archive/GPM28700000061.xml; L745A, http://human.thegpm.org/thegpm-cgi/plist.pl?path=/tandem/archive/GPM28700000059.xml. The mass spectrometry proteomics data have been deposited in the ProteomeXchange Consortium via the PRIDE (61) partner repository with the data set identifier PXD004924.

### Confocal fluorescence microscopy.

Cells grown to mid-exponential growth phase in SD medium, lacking leucine and uracil and supplemented with 100 mg/liter adenine and 100 mg/liter l-tryptophan, were concentrated ∼60-fold in SD medium lacking leucine and uracil and supplemented with 100 mg/liter adenine and 100 mg/liter l-tryptophan before addition of 2 mM DTT. Five microliters of untreated cell suspensions or cell suspensions exposed to 2 mM DTT were examined on a Zeiss LSM 880 with Airyscan confocal inverted microscope (Carl Zeiss Ltd., Cambridge, United Kingdom) using a Plan-Apochromat 63×/1.4 oil differential interference contrast (DIC) M27 objective and an MDS 488/594 beam splitter. GFP fluorescence was excited with a 488-nm laser at a power setting of 5 to 8%, and its fluorescence emission was collected between 498 and 564 nm with a photomultiplier tube. mCherry fluorescence was excited with a 594-nm laser at a power setting of 28 to 40%, and its fluorescence emission was collected at 694 to 754 nm with a gallium arsenide phosphide detector. Gain settings between 650 and 900 were used to collect images.

### Statistical analyses.

Experimental data are presented as means and standard errors. For composite parameters, errors were propagated using the law of error propagation for random, independent errors (62). All data were analyzed for normality using the D'Agostino-Pearson omnibus normality test (63), equality of variances (homoscedasticity) using the Brown-Forsythe test (64) or Bartlett's test (65), and, for additivity of means, treatment effects, and errors, using Tukey's test (66, 67) before ANOVA. Heteroscedastic or nonadditive data were ln transformed before ANOVA (66) or analyzed by Welch's test (68) followed by the Games-Howell *post hoc* test (69). Normality was examined on the pooled data for the *ire1*Δ deletion strain transformed with YCplac33 or, alternatively, on all pooled data points before induction of ER stress. In all analyses, a *P* value of ≤0.05 was considered statistically significant. Brown-Forsythe tests for equality of variances, Tukey's test for additivity, Welch's tests, and Games-Howell *post hoc* tests were performed in Excel 2010 (Microsoft Corporation, Redmond, WA, USA) using the Real Statistics plug-in for Microsoft Excel (http://www.real-statistics.com/). All other statistical calculations were performed in Prism 6.07 (GraphPad Software, La Jolla, CA).
